# Morphomolecular identification and considerations of the infestation site adaptations of *Pricea multae* (Thoracocotylidae: Priceinae) from *Scomberomorus commerson*, off Arabian Gulf, Saudi Arabia

**DOI:** 10.1590/S1984-29612022041

**Published:** 2022-08-01

**Authors:** Hanadi Baker Baghdadi, Afaf Abdullah Mubark Al-Salem, Mustafa Mohamed Ibrahim, Abdelgayed Metwali Younes, Salama Mostafa Aboelenin, Elsayed Mahmoud Bayoumy

**Affiliations:** 1 Biology Department, College of Science, Imam Abdulrahman Bin Faisal University, Dammam City, Saudi Arabia; 2 Basic and Applied Scientific Research Center, Imam Abdulrahman Bin Faisal University, Dammam, Saudi Arabia; 3 Animal Health Research Institue, Dokki ,Giza, 12618, Egypt.; 4 Hydrobiology Department, National Research Centre, Dokki, Egypt; 5 Biology Department, Turabah University College, Taif University, Taif, Saudi Arabia

**Keywords:** Neothoracocotylidae, priceinae, Scomberomorus commerson, taxonomy integrative, Neotoracocotylidae, priceinae, Scomberomorus Commerson, taxonomia integrativa

## Abstract

Monogeneans *Pricea multae* naturally infested 42 of the 120 (35%) mackerel fish (*Scomberomorus commerson*) examined. For the first time, an infestation was discovered off the coast of Jubil in the Arabian Gulf of Saudi Arabia. Based on the structure clarified through light and electron microscopy of mounted specimens and molecular analysis of rDNA and measurements of this monogenean parasite was identified as *P. multae*. The tegumental surface of the parasite was characterized by tegumental ridges running transversally, generating folds in both the dorsal and ventral surfaces of the body at regular intervals. The study clarified the importance and function of the micro-structures, such as tegumental folds, perforations, sensory ganglia present on the parasite's surface, and the larger hamulus supported by a relatively unmodified internal spine. This monogenean parasite has adapted to its host infestation site uniquely.

## Resumo

Monogenéticos *Pricea multae* infestaram naturalmente 42 dos 120 (35%) peixes cavala (*Scomberomorus commerson*) examinados. Pela primeira vez, uma infestação foi descoberta na costa de Jubil, no Golfo Árabe, Arábia Saudita. Com base no entendimento de suas estruturas, por meio da microscopia de luz e de varredura eletrônica em amostras analisadas pela biologia molecular do rDNA e as medições, esse parasito monogenético foi identificado como *P. multae*. A superfície tegumental do parasito foi caracterizada por cristas tegumentais dispostas transversalmente, gerando dobras nas superfícies dorsal e ventral do corpo em intervalos regulares. O estudo esclareceu a importância e a função das microestruturas, como dobras e perfurações no tegumento, gânglios sensoriais que estavam presentes na superfície do parasito e o hâmulo maior apoiado por uma coluna interna relativamente não modificada. Esse parasito monogenético se adaptou exclusivamente ao local de infestação no hospedeiro.

## Introduction

Since the earliest times, fish have played an important role in the aquatic ecosystem and represent the main source of income for many countries, especially with great economic value (Mehanna, 2022). Presently, great consideration is paid to fish production as the replicable source of protein (Waite et al., 2014; Ganguly et al., 2022). Epipelagic and migratory marine fish, particularly those belonging to Scombridae fish, include species of high commercial interest. *Scomberomorus commerson* (Scombridae) is a predatory marine and one of the most expensive and high-quality fish due to its nutritious proteins (Claereboudt et al., 2005; Rajesh et al., 2017, Thai et al., 2021).

Few and sporadic investigations spotlight fish parasites in the Arabian Gulf, which are more scarce than recorded on it, specially monogenean parasites (Bayoumy et al., 2012). The monogenean parasites are mainly external parasites infesting aquatic vertebrates, especially fish, where a few genera are endoparasites (Bayoumy et al., 2015; Klapper et al., 2017). Anemia and potential host mortality are primarily caused by the infestation of monogeneans (Ramasamy & Hanna, 1985; Woo & Bruno, 2011; Rigos et al., 2021). The Neothoracocotylidae classification includes Neothoracocotylinae Lebedev, 1969; Priceinae Chauhan, 1953; Scomberomorocotylinae; and Thoracocotylinae Price, 1936 (Rohde & Hayward, 1999a, b). Furthermore, unlike other Neothoracocotylidae subfamilies, Priceinae has one or two rows of clamps, two pairs of large hamuli, and few male copulatory spines (Rohde & Hayward, 1999a, b).

*Pricea multae* (Neothoracocotylidae: Priceinae) was first recorded in the Indian Ocean as isolated from the gills of *Cybium lanceolatus,* and later; it was reported as present in the gills of several species of mackerel at different localities in the Indian Ocean, off the Australian coast and the South China Sea (Ramasamy et al., 1986, Rohde & Hayward, 1999a, b). For a short time, it has an optional intermediate host (Rohde & Hayward, 1999a, b).

In this investigation, we are concerned not only with the morpho-molecular description through light and scan electron microscopes of *P. multae* from the gills of *S. commerson* in the Arabian Gulf, off Jubail, but also with spotlighting on adaptations of this monogenean species.

## Materials and Methods

### Fish samples

Freshly caught *S. commerson* ('Spanish mackerel) samples (N=120) were collected weekly of local name: 'Kanad' from local fishermen along the Arabian Gulf, off Jubail, Saudi Arabia ('27 ° 57.9” N and '49° 40' 43.4” E) during the period from May 1^st^, to August 31^st,^ 2018 and were brought to the laboratory of the Faculty of Science wrapped in ice bags. Fish used for parasite collection were dead, been commercially caught, and available for purchase at fish markets. Dissecting fish has complied with all relevant regulations.

### Ethics statement

The fish used to collect the parasites were dead, commercially caught, and available on the fish market. All relevant regulations were observed when dissecting fish.

### Parasitological examination

#### Light microscopy

After separating the gills, they were placed in separate Petri dishes with seawater and examined for the presence of monogeneans using an Olympus SZ61 stereomicroscope. The monogeneans were examined alive or after being fixed in 70% alcohol under a coverslip. Others were preserved in 70% ethanol after being fixed in 4% formalin. After that, they were stained with Mayer's carmine and dehydrated with ethanol. Clove oil was used to clear dehydrated specimens before mounting them in gelatin-glycerol media. A light microscope was used to examine and photograph mounted specimens (Olympus CH40). Illustrations of stained specimens were created using a previous microscope with a drawing tube, then scanned and redrawn using Photoshop software on a computer. A few specimens (N=20) were mounted in ammonium picrate glycerin medium to note details of the hard parts of the haptor and the organization of the terminal genitalia. Measurements were taken with an ocular micrometer on flattened stained specimens and are presented as the mean (± St. error) followed by the range mean in parentheses. The opisthohaptor is included in the body length.

#### Scanning electron microscopy (SEM)

Some isolated monogeneans specimens were fixed in a 4% aqueous glutaraldehyde solution (4°C for 48 Hrs.). They were then washed thoroughly with cacodylate buffer and post-fixed for 4 Hrs. with aqueous osmium tetroxide (Os O4) and dehydrated through alcohol. Later, they were dried in a Tousimis Autosamdri – 815 Coater, E300 critical point drying apparatus using liquid CO_2_. The specimens were whole-mounted on an aluminum stub and fixed by a double-phase sticker. The specimens were then coated with gold-palladium in a Sputter Coating Evaporator unit (S.P.I. Model-Sputter Carbon/ Gold Coater) (Bayoumy et al., 2006). The specimens were examined using a JEOL JEM-2100 scanning electron microscope operating at 20 Kev. All the preparations for SEM were done at Electron Microscopy Unit at Mansoura University.

Molecular procedures for molecular identification, one specimen preserved in 70% ethanol was rinsed in ultrapure water. According to the manufacturer's procedure, the Genomic DNA. Purification Kit (Invitrogen, USA) was used to extract total genomic DNA. The primers C1 (5′-ACCCGCTGAATTTAAGCAT-3′) and D2 (5′-TGGTCCGTTTCAAGAC-3′) were used to amplify DNA. specific to the D1-D2 domain of the large subunit region of the 28S ribosomal gene (28S rRNA) (Hassouna et al., 1984). DreamTaq PCR master mix (Thermo Scientific) was used for PCR amplification, with an initial denaturation at 94°C for 3 minutes, followed by 35 cycles of 30 seconds at 93°C, 30 seconds at 58°C, and 90 seconds at 72°C, with a final 7-minute extension step at 72°C.

The PCR product was purified using the Purelink^TM^ Quick Gel Purification Kit (Invitrogen, USA.) and submitted straight for sequencing on a 3730xl DNA sequencer (Applied Biosystems, USA) using the identical primers stated above. The nucleotide sequence has been submitted to GenBank. The nucleotide sequence acquired in this research was aligned with prior sequences submitted in GenBank using BLASTn (http://www.ncbi.nlm.nih.gov/blast).

MEGAX software was used to infer phylogenetic relationships based on incomplete 28S rRNA sequences using the maximum likelihood and the neighbor-joining methods. The trees' credibility was evaluated using the bootstrap technique with 1000 replicates, and the divergence time was calculated using the Tamura and Nei model.

## Results

### Taxonomic Summary

Host: *Scomberomorus commerson*.

Site of infestation: gills.

Locality: Arabian Gulf, Saudi Arabia.

Prevalence: 35%. (42 of 120 examined fish)

Range of infection: 1-5.

**Figure 1:**
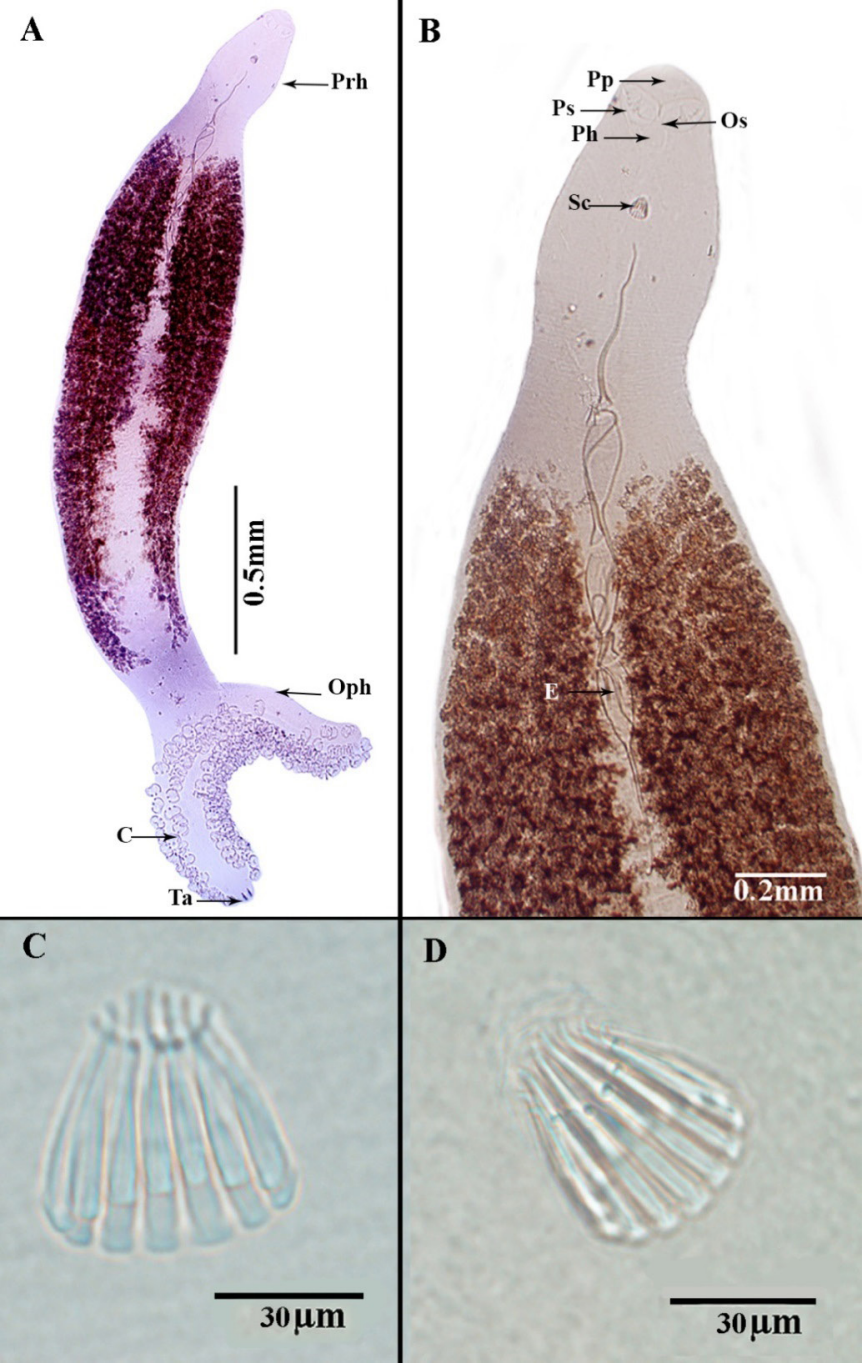
Photomicrograph of *Pricea multae*. (A) hole-mount, ventral view; (B) Anterior extremity showing the relative position of prohaptoral suckers, pharynx, and spinose male copulatory organ in genital atrium and cirrus (C) Lateral view showing the spines of male copulatory, penis invaginated; (D) Lateral view showing the spines of male copulatory (**abbreviations**: C: clamp; E: egg; Ph: pharynx; Pp: pre-oral pits; Prh: prohaptor; Oph: opisthohaptor; Ps: Pre-oral sucker; Ta: Terminal anchors; and Sc: sclerites of copulatory organ).

**Figure 2:**
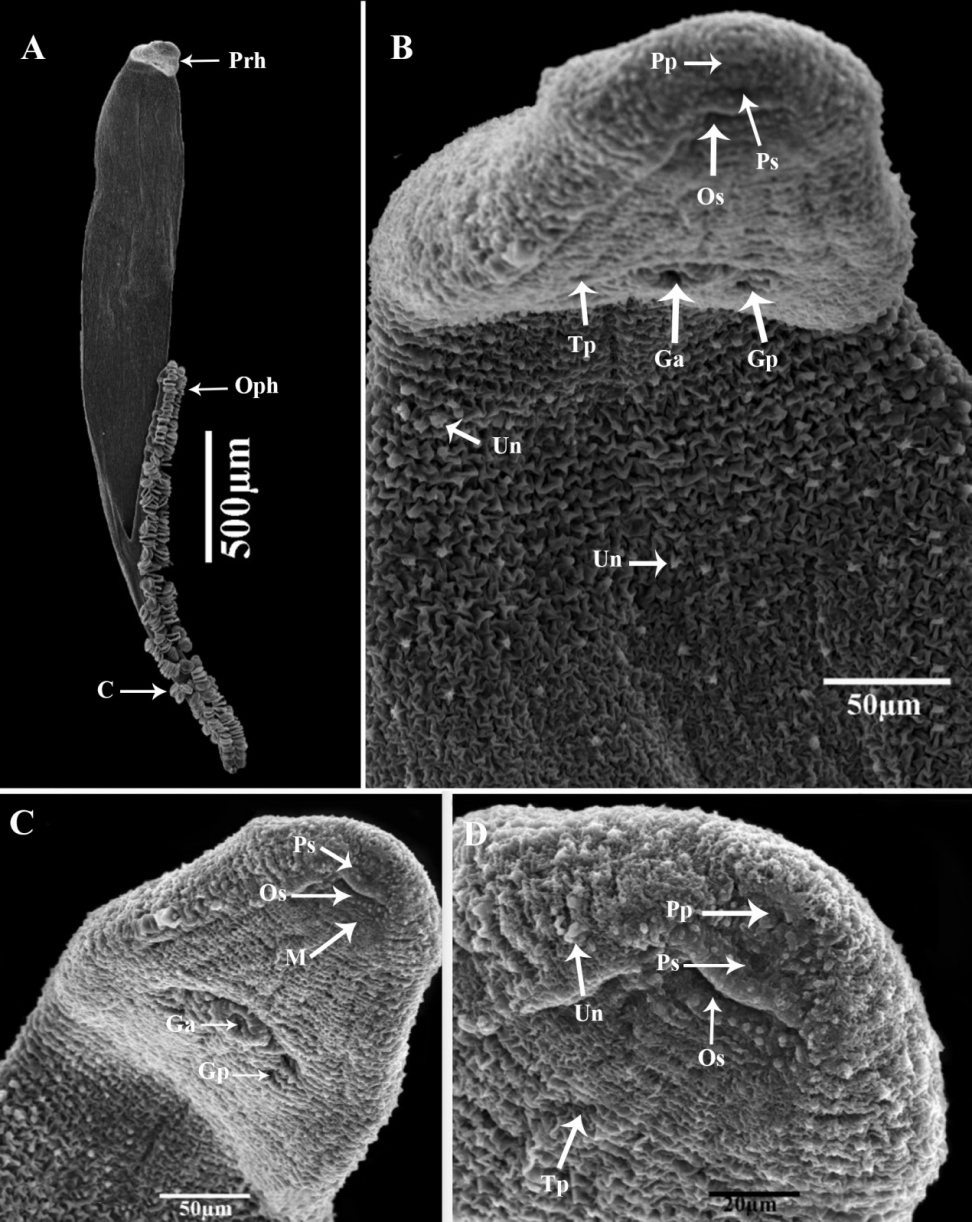
Scanning electron micrographs of *Pricea multae*. (A) General view of the ventral surface (note: one row of many clamps on each side of the body) (B) General view of prohaptor (note: the uni-ciliated sensory ending, the pits, and folds of the tegumental surface; (C&D) Anterior region showing the position of the pre-oral pits, the oral sucker and genital atrium (note the presence of many vaginal pores around the genital atrium). (**abbreviations**: C: clamp; Ga: genital atrium; Gp: genital pore; M: mouth; Pp: pre-oral pits; Ps: pre-oral uniciliated sensilia; Prh: prohaptor; Oph: opithohaptur; Os: oral sucker; Tp: tegumental pores; and Un: Uniciliated sensory).

Description: (Based on 20 whole mounted specimens.) A total of 132 parasite specimens were collected from the sampled mackerel fish Body long, smooth, curving to the right, flattened dorso-ventrally, and pointed to the anterior end and the opisthaptor bifurcate (Figures 1A and 2A). The fore and hind bodies with tegumental corrugations on dorsal and ventrolateral surfaces. The tegumental ridges run transversally, generating folds in dorsal and ventral surfaces of the body at regular intervals (Figure 2B). Pit-like depressions, as well as microvillus-like tegumental projections, between the folds, densely packed with papillary-like sensory endings that are uni-ciliated (Figures 2C and 2D). The total body length of compressed specimens was 2900 (±210), (2750-3940) in the uncompressed 2700 (±120), (2530-3240).

**Figure 3:**
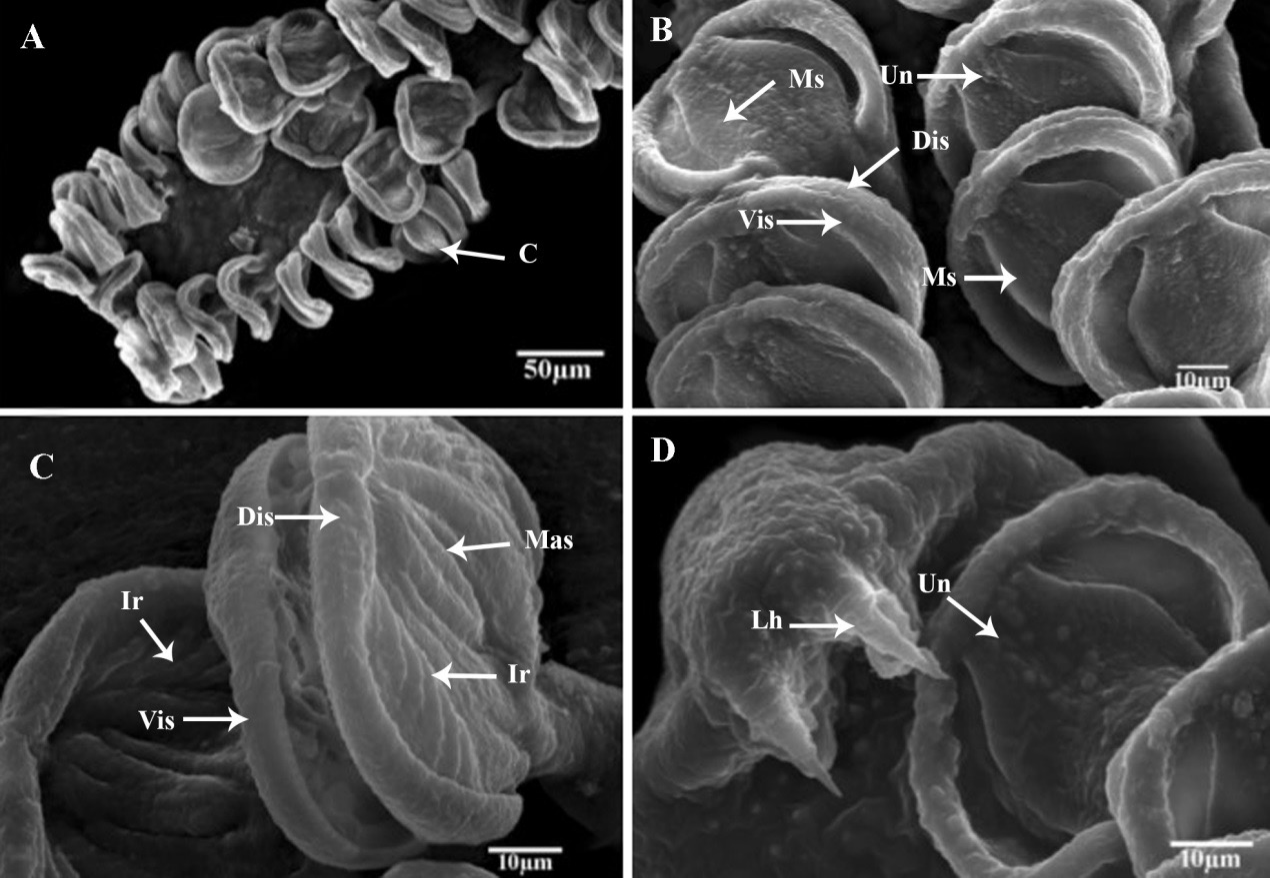
Scanning electron micrographs *Pricea multae*. (A) General view of Posterior lappet; (B) Posterior lappet; C&D Clamps and large marginal hamulus (**abbreviations**: Ac: accessory collar; Bas: basal accessory sclerite; C: clamp; Dis: dorsal inner sclerite; Ir: lateral ribs; Lh: lateral hamulus; Mas: median accessory sclerite; Ms: median sclerite; Un: Uniciliated sensory; and Vis: ventral internal sclerite).

Anterior body length with 2450 (±120), (2250-2630). The average width at the germarium level 520 (±110), (420-650). The prohaptor with two septate, sub-terminal, sub-oval, and ventral buccal cavities. The buccal cavity was oval than circular and surrounded by a pre-oral pit (20 ± 0.8× 17±0.6) 18-22×16-19 length by width and 20-20 uni-ciliated sensory endings (Figure 1B). Mouth subterminal, ventral, approximately small and surrounded by sensory receptors (Figure 3A). Each one has knob-like cilium. Pharynx small, bulb-shaped, muscular, and sub-spherical 58 (±1.2), (54-62). The esophagus was short without diverticula. Intestine bifurcation behind the level of the vaginal opening; caeca with medial and lateral diverticula unbranched, extending into posterior body region but not into opisthohaptor and not confluent posteriorly. On each side of the body, note the two-row of 45(±2) (38-52) clamps with a maximum width of 49(±0.11), (46-53), and the brown or black pigment (haematin) derived from ingested host blood. Only the ventral surface of the haptor has non-ciliated dome-shaped sensory endings. The haptor's dorsal and lateral surfaces smooth.

**Figure 4:**
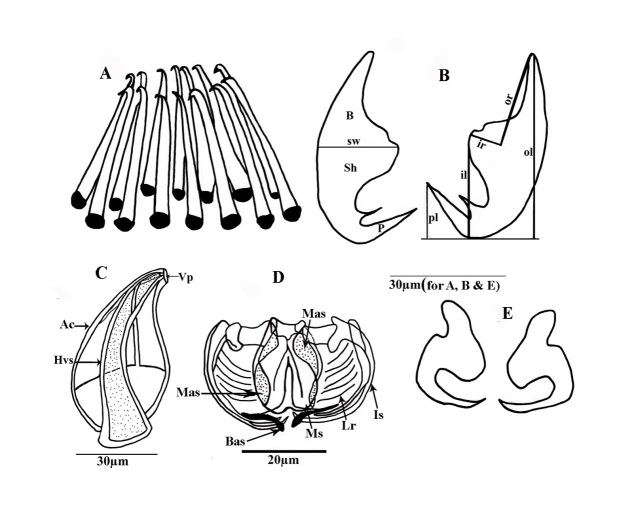
Diagram of *Pricea multae* showing hard parts of the clamp, hamulus, and genitalia structures (A) Corona of spines of the male copulatory organ; (B) Large hamulus of terminal lappet Posterior lappet, ventral view; (C) Vaginal spine with apical collar (D) Clamp structure; (E) Small hamulus of terminal lappet (**abbreviations**: Ac: accessory collar; B: blade; Bas: basal accessory sclerite; Hvs: hallow conical spin; il: total inner length; ir: inner root length; ls: lateral sclerite; Mas: median accessory sclerite; lr: lateral ribs; Ms: median sclerite; ol: total length; or: outer root length; pl: point length; Sh: shaft; P: point; sw: shaft width; and Vp: vaginal pore).

**Figure 5:**
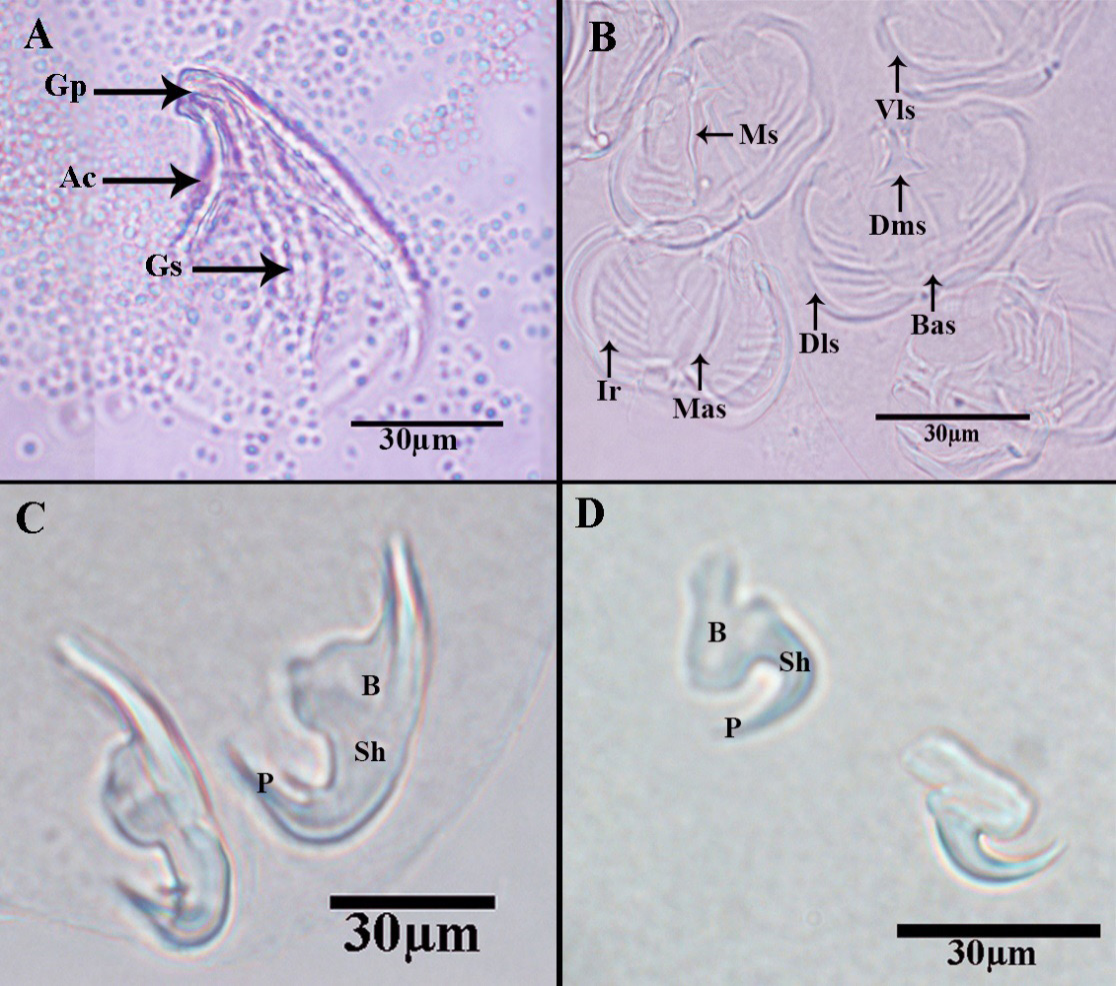
Photomicrograph of *Pricea multae*. (A) Vaginal spine with apical collar; (B) Posterior lappet, ventral view; (C) Large hamulus of terminal lappet; (D) Small hmuluas of terminal lappet (**abbreviations**: Ac: accessory collar; B: blade; Bas: basal accessory sclerite; Dms: dorsal median sclerite; Dls: dorsal lateral sclerite; Gp: genital pore; Gs: genital spine; Ir: lateral ribs; Mas: median accessory sclerite; Ms: median sclerite; P: point; Sh: shaft; and Vls: Ventral lateral sclerite).

The testes, 20 in number in the median field, arranged in two rows from behind the ovary to between the end of the caeca and the opisthaptor. Cirrus a circle of 14 (13-15) copulatory spines with a total length of 42.8 (±0.13), (41-44) (Figures 1C;1D and 4A). Gonopore a short distance behind the pharynx, approximately at the level of excretory openings, genital atrium unarmed. The germinal part of ovary is heavily branched, with a long coiled ovarian loop pointing anteriorly and germinal and terminal prats pointing posteriorly . Small follicular vitellaria in the lateral fields from the caecal bifurcation to the end of the caeca. The vaginal opening is armed with the sclerotized, hallow conical spine with an apical collar, in front of the caecal bifurcation. Egg of 580 (620-575; n = 2) in length; ends with long bipolar filaments (Figures 4C and 5A).

Sclerites of clamps consisted of one median U-shaped piece (Ms: median sclerite) bifurcated at each end, two anterolateral curved pieces (Dls: dorsal lateral sclerite), two broader pieces external (Mas: median accessory sclerite), and posterior to curved lateral sclerites and two basal accessory pieces. Inner walls of clamp structure with approximately 10(8-12) muscular lateral ribs (Ir) Median clamp sclerites with length of 19.8 (±0.25), (19.6-21.7) (Figures 3B and 3C). The median accessory sclerite length 17.9 (± 0.3), (17.4-18.5). The dorsolateral clamp sclerite length 45 (±3), (41-52), and the basal accessory sclerite 10 (±1), (8 -12) (Figures 4D and 5B).

Large hamulus of terminal lappet well-developed outer root bifid recurved points and minute accessory spine at the inner margin (Figures 3D; 4E and 5D); outer length 54.3 (±0.14), (51-57); inner length 28.6 (±0.12) (27-31); outer root 28.6 (±0.11), (26-30); shaft width 24.3 (±0.14), (22-25) and inner root length 11.4 (± 0.04), (9-12) and the point 5(±0.21) (4-6). Small hamulus of terminal lappet without roots (Figures 4B and 5C); outer with length of 28.6 (±0.12), (26-30) and shaft with width of 24.2 (± 0.13), (0.23-26).

### Molecular identification

The nucleotide sequence has been submitted to GenBank under the accession number ON032998. This isolate sequence was identical to previously deposited *P. multae* isolates from Israel (accession numbers MT995137, MT995138, MT995139, MT995140) (Direct submission in 2020). Furthermore, this isolate has the highest resemblance (98.81%) to the specimen available for *P. multae* (accession number AF026111) collected from *S. commerson* from Heron Island, Australia.

**Figure 6:**
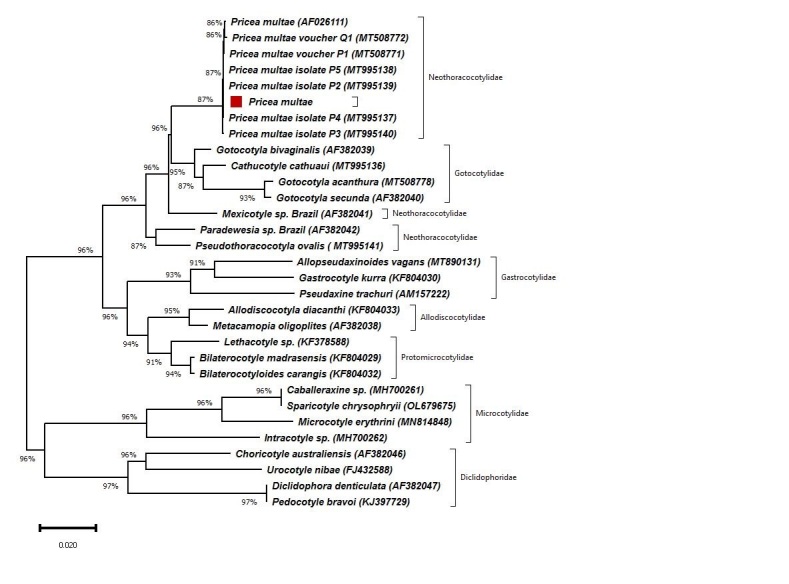
Phylogenetic tree based on the 28S rRNA sequence of the Monogenea using the maximum likelihood method. The *Pricea multae* isolated in the present study is indicated with a red square.

Our phylogenies inferred using the maximum likelihood and the neighbor-joining method produced similar topologies. The maximum likelihood topology is shown in Figure 6. A phylogenetic analysis of 28S sequences revealed that the *Pricea multae* reported in this work belongs to the same clade as other *Pricea multae* isolates, *Mexicotyle* sp. and *Paradewesia* sp. (Neothoracocotylidae family), as well as *Gotocotyla secunda* and *Gotocotyla bivaginalis* (Gotocotylidae family). Furthermore, this isolate and *P. multae* isolates were the dataset's most closely related Neothoracocotylidae.

## Discussion

Monogeneans are pathogenic parasites that live in a single host and are found in wild and captive fish. Priceinae members have been found in the gills of scombrid fishes of the genus *Scomberomorus* (and possibly Acanthocybium, Rastrelliger, and Katsuwonus) from warm to warm temperate seas around the world (Rohde & Hayward, 1999a). The parasites under investigation were previously recorded on the Shina sea, the Indian Ocean, and the Australian east coasts (Rohde, 1976; Rohde & Hayward, 1999a, b). The widespread of this monogenean species (*P. multae*) may be attributed to the wide range of the geographical distribution of their host (*S. commerson*) (Rohde, 1976). It is the first occurrence of the monogenean *P. multae* parasitizing *S. commerson* off Arabian Gulf, Saudi Arabia.

According to Rohde & Hayward (1999a, b), Priceinae members are characterized by one or two rows of clamps, two pairs of large hamuli, and a small number of male copulatory spines. Small hamuli of the present material coexist with large hamuli on the terminal haptor lappet rather than in the typical prehaptoral position (Gupta & Chanana, 1976; Hayward & Rohde, 1999). So, the material under discussion has all Priceinae characteristic features. Comparing the morphometric data for the present material with the redescription of ‎this species) Rohde, 1976; Lebedev, 1986; Rohde & Hayward, 1999a, b) reveals a few differences (Table 1). Some structures of present specimens were not measured in detail, such as ‎the copulatory spine length, clamp parts, and hamuli measurements.

**Table 1:** Comparison between the present material (*Pricea multae*) and previous described species from deferent *Scomberomorus* species (All measurements in µ).

(Rohde & Hayward, 1999a)	(Rohde & Hayward, 1999a)	(Rohde & Hayward, 1999a)	(Lebedev, 1986)	(Rohde, 1976)	Present study	Aspect
*S. koreanus*	*S. lineolatus*	*S. commerson*	*S. commerson* and *S. guttatus*	*S. commerson* and *S. queenslandicus*	*S. commerson*	Host species
1,580-3,480 (2,580)	2,490-5,140 (3,500)	1,740-4,580 (2,940)	2,500-6,700	3,300-6,100 (5,040)	2900 (±210), (2750-3940)	Total body length
-	-	-	-	-	2450 (±120), (2250-2630)	Anterior body length
-	-	-	-	-	520 (±110), (420-650)	Maximum body width
				-	20 ± 0.8× 17±0.6) 18-22×16-19	Preoral pit size
-	-	-	-	-	58 (±1.2), (54-62)	Pharynx length
5–14 (13)	13-15 (14)	7-15 (13)	12-14	12-17 (14)	14 (13-15)	No. copulatory spines
-	-	-	-	-	42.8 (±0.13), (41-44)	Copulatory spines length
62	60-68 (64)	58-70 (64)	52-73	-	61(±3)(56-69)	Vaginal spine length
1,040-1,770 (1,470)	1,010-1,830 (1,410)	660-2,750 (1,790)	1,690-1,910	1,100-2,600 (2,190)	1450 (±2)(1390-1545)	Haptor length
41-51	12-54	33-78	46-90	41-89	45(±2) (38-52)	No. clamp pairs
59-72 (66)	68-91 (82)	70-84 (76)	70-100	79-105 (95)	49(±0.11), (46-53)	Maximum clamp width
-	-	-	-	-	19.8 (±0.25), (19.6-21.7)	Median clamp sclerites length
-	-	-	-	-	17.9 (± 0.3), (17.4-18.5)	Median accessory sclerite length
-	-	-	-	-	45 (±3), (41-52) 4	Dorso-lateral clamp sclerite length
-	-	-	-	-	10 (±1), (8-12)	Basal accessory sclerite length
	5-8	6-7	‘many’	7-8	10(8-12)	No. clamp ribs
Large hannuli:						
49-53 (51)	50-60 (56)	54-58 (56)	40-65	44-67 (56)	54.3 (±0.14), (51-57)	outer length
-	-	-	-	-	28.6 (±0.12) (27-31)	inner length
-	-	-	-	-	28.6 (±0.11), (26-30)	outer root
-	-	-	-	-	11.4 (± 0.04), (9-12)	inner root
-	-	-	-	-	24.3 (±0.14), (22-25)	shaft width
-	-	-	-	-	5(±0.21) (4-6)	the point
-	-	-	-	-		Small hamulus:
	21-30	-	22-30	-	28.6 (±0.12), (26-30)	outer length
-	-	-	-	-	24.2 (± 0.13), (0.23-26)	shaft width

This study showed that this isolate and *P. multae* isolates (accession numbers MT995137, MT995138, MT995139, MT995140, and AF026111) were the dataset's most closely related Neothoracocotylidae. The phylogenetic analysis of 28S sequences showed that the species forms a clade with members of the family Neothoracocotylidae and the family Gotocotylidae. Similar to these results (Olson & Littlewood, 2002) found that the oligonchoinean families, Gotocotylidae and Neothoracocotylidae, were paraphyletic.

Despite the importance of confirming the taxonomic status of parasite, one of the goals of this study was to learn more about its functional appropriateness and the significance of its microstructures. The observations indicate that this monogenean species has a cylindrical body with multiple clamps and a pair of hamulus through which attachment to the gills of the host is maintained. As a result, numerous tegument folds and perforations enhance surface area and facilitate absorption and excretion. The sensory ganglia present on the surface of parasite serve as sensory organs for detecting the host type, site of infestation, and water quality. The papilla-like uni-ciliated sensory endings found all over the body surface could be rheo- or tango-receptors. In contrast, the sensory endings around the oral apertures are thought to be involved with feeding (Hadied et al., 2015). Pits, folds, and a few scattered microvilli-like structures on the body surface may help with nutrient absorption, osmoregulation, and respiratory gaseous exchange (Ramasamy et al., 1986; Thompson & Geary, 2003). Moreover, the haptor's non-ciliated sensory structures may help the fluke attach to the host's gills (Ramasamy & Brennan, 2000; Hadied et al., 2015). The posterior oriented curved hamulus spines are considered the primary attachment organ as they are highly modified, whereas a relatively unmodified internal spine supports the larger one.
